# Photosynthetic contribution of the ear to grain filling in wheat: a comparison of different methodologies for evaluation

**DOI:** 10.1093/jxb/erw116

**Published:** 2016-03-24

**Authors:** Rut Sanchez-Bragado, Gemma Molero, Matthew P. Reynolds, Jose Luis Araus

**Affiliations:** ^1^Unitat de Fisiologia Vegetal, Facultat de Biologia, Universitat de Barcelona, Diagonal 643, Spain; ^2^International Maize and Wheat Improvement Center (CIMMYT), El Batán, Texcoco CP 56130, Mexico

**Keywords:** Carbon isotope composition, culm, ear, grain filling, photosynthesis, wheat.

## Abstract

The culm (particularly the flag leaf) and the ear are believed to play a major role in providing assimilates for grain filling in wheat. However, the results obtained in the past varied depending on the methodology applied. Three different methodologies were compared that aimed to assess the relative contribution of the culm (photosynthetic organs below the ear) and the ear to grain filling. The first two consisted of applications of photosynthesis inhibition treatments, including the use of the herbicide DCMU and organ shading. The third was a non-intrusive method that compared the carbon isotope composition (δ^13^C) of mature kernels with the δ^13^C of the water-soluble fraction of the peduncle, awns and glumes. Several advanced CIMMYT lines were tested under good agronomic conditions. The δ^13^C approach assigned a higher photosynthetic contribution to the ear than to the culm. However, some methodological considerations should be taken into account when applying the δ^13^C approach, particularly the sampling method used, in order to prevent post-harvest respiration. The shading approach assigned a similar contribution to the ear as to the culm. The DCMU approach assigned a greater role to the culm but herbicide application to the culm affected the ear, thus biasing the final grain weight. Moreover DCMU and shading approaches may cause compensatory effects which overestimated the contribution of unaffected organs. This study may help to develop precise phenotyping tools to identify physiological traits such as ear photosynthesis that could contribute towards increasing grain yield.

## Highlight

This study proves the key role of the ear as a source of assimilates for grain filling under good agronomical conditions and compares different methodologies of phenotyping.

## Introduction

Whereas breeding efforts in recent decades have been focused on improving crop adaptation to disease and abiotic stresses (Araus *et al.*, 2002), interest in raising the yield potential has grown only recently (Reynolds *et al.*, 2012). Although wheat breeding programmes still achieve steady genetic gains (Manès *et al.*, 2012; Sharma *et al.*, 2012), there is a need to develop more efficient wheat breeding methodologies that require less time and resources and complement existing (traditional) breeding techniques (Araus *et al.*, 2008). Part of the attributes responsible for yield gains in wheat productivity may be related to biomass partitioning to reproductive organs (Austin *et al.*, 1989). The increase in sink strength and harvest index due to dwarfism alleles (Maydup *et al.*, 2012), or the increase in the ‘communalism’ habit of the crop canopy (Reynolds *et al.*, 1994) in order to avoid the evolutionary strategy that minimized the effect of herbivory and competition for light and resources from plants within a canopy (Reynolds *et al.*, 2009), has led to genetic gains in grain yield. One of the breeding techniques proposed to increase yield potential and improve the adaptation to the increasing incidence of abiotic stresses (such as drought and heat) due to climate change is to select for higher ear photosynthesis (Tambussi *et al.*, 2005, 2007*b*; Araus *et al.*, 2008). Hence, ear photosynthesis is thought to play an important role in terms of the source of photoassimilates during grain filling, not only under drought, but also under good agronomical conditions (Araus *et al.*, 1993; Tambussi *et al.*, 2005, 2007*b*; Maydup *et al.*, 2010; Sanchez-Bragado *et al.*, 2014*b*). Although under good agronomical conditions the actual photosynthetic source is often in excess of the sink (Slafer and Savin, 1994; Borrás *et al.*, 2004), recent evidence indicates that limitations to the source (Álvaro *et al.*, 2008) rather than the sink (Slafer *et al.*, 1999) have been emerging in modern cultivars of wheat. In addition, it is widely reported that different fungal diseases may affect leaves (Robert *et al.*, 2005) more than ears (Tiedemann and Firsching, 2000). Therefore, in conditions where leaf photosynthesis is limited, assessing the photosynthetic contribution of the ear to grain yield may be relevant.

Assimilates transported to the grain during grain filling in C_3_ cereals are mainly provided by three sources: (i) flag leaf (blade and sheath) photosynthesis (Evans *et al.*, 1975); (ii) pre-anthesis reserves (Gebbing and Schnyder, 1999); and (iii) ear photosynthesis (Tambussi *et al.*, 2007*b*). However, the proportion in terms of the contribution of assimilates to grain filling of each of the three mentioned sources still remains imperfectly known due to methodological constraints (Evans *et al.*, 1975; Nicolas and Turner, 1993; Tambussi *et al.*, 2007*a*). Such methodological limitations are closely related to the quantification and separation of the ear photosynthesis from assimilates that come from the leaves and are retranslocated during grain filling. In point of fact, compared with the leaves, the photosynthetic contribution of ears has been less studied and still remains unclear, particularly under field conditions (Maydup *et al.*, 2014).

Thus, alternative approaches to solve such methodological constraints have been deployed to evaluate the ear contribution to grain filling (Borrás *et al.*, 2004; Maydup *et al.*, 2010). The most commonly used approaches include detachment (i.e. organ-specific) of some plant parts, such as stem defoliation at the anthesis stage (Ahmadi *et al.*, 2009); inhibition of photosynthesis based on shading (Aggarwal *et al.*, 1990; Araus *et al.*, 1993); application of herbicides (Maydup *et al.* 2010); or desiccant treatments (Blum *et al.*, 1983; Nicolas and Turner, 1993; Saeidi *et al.*, 2012). Nevertheless, these approaches do not exempt organs from being affected by physiological processes other than photosynthesis (Tambussi *et al.*, 2007*a*), such as respiration, ripening, etc (Kriedemann, 1966) that hypothetically may bias the final grain weight. Furthermore, an additional source of variation in growing grains may be related to remobilization of stem reserves due to a decrease in photoassimilate production after anthesis (Chanishvili *et al.*, 2005). Nonetheless, such remobilization has been observed to begin only when the maximum fill rate of the grains cannot be maintained by the current photosynthesis (Bingham *et al.*, 2007; Slewinski, 2012). Likewise, the potential contribution of stem reserves during grain filling under good agronomical conditions seems to be low because the photosynthetic capacity of plants during grain filling exceeds the sink demand of growing grains (Slafer and Andrade, 1991; Dreccer *et al.*, 2009).

Therefore, use of the stable carbon isotope signature in its natural abundance (δ^13^C) may help to elucidate the relative contribution of the different photosynthetic organs with the added advantage of being a non-intrusive approach (Sanchez-Bragado *et al.*, 2014*a*, *b*). Moreover the novel approach using δ^13^C in its natural abundance may help to avoid unwanted compensatory effects triggered by intrusive methods (Chanishvili *et al.*, 2005).

The main objective of this work was to compare different experimental approaches aiming to assess the relative contribution of ear photosynthesis and the rest of the plant to grain filling. The study was performed in a set of high-yielding advanced lines of bread wheat from CIMMYT (International Maize and Wheat Improvement Center) growing under well-managed agronomic conditions. Three different techniques were used: inhibition of ear and culm photosynthesis through (i) herbicide DCMU application or (ii) by shading each organ, and (iii) the analysis of the δ^13^C of assimilates from different plant parts (awns and peduncle) as a criterion to assess in a non-disturbing manner the relative contribution of ear and culm photosynthesis to grain filling. In such a way the δ^13^C of assimilates from the awns and peduncles were analysed around the mid stage of grain filling.

## Materials and methods

### Germplasm used and experimental conditions

Six advanced bread wheat (*Triticum aestivum* L.) lines with similar phenology, from the CIMCOG (CIMMYT Mexico Core Germplasm) panel were selected. The field experiments were conducted during the spring growing seasons of 2012 and 2013 at CIMMYT’s Experimental Station, Norman E. Borlaug (CENEB), near Ciudad Obregón, Mexico (27°24ʹ N, 109°56ʹ W, 38 m asl), under fully irrigated conditions. The experimental design was a randomized lattice with three replications in 8.5 m long plots as explained elsewhere (Sanchez-Bragado *et al.*, 2014*b*). Experiments were sown on 9 December 2011 and 23 November 2012, and immediately irrigated to promote germination. The emergence dates were 16 and 2 December of 2011 and 2012, respectively. Environmental conditions during the growing seasons are detailed in Supplementary Fig. S1 at *JXB* online. Harvesting was performed by machine on 15 May 2012 and manually on 6–7 May 2013, respectively.

### Agronomic traits

For each plot, yield components were determined in ~5.7 m^2^ using standard protocols (Pask *et al.*, 2012). In addition, phenology was recorded throughout the cycle (Zadoks *et al.*, 1974).

### Leaf and ear photosynthesis and respiration

Photosynthetic and respiration rates of the flag leaf blade and the ear were measured during both seasons (2012 and 2013) as carbon uptake using a LI-6400XT portable gas exchange photosynthesis system (Li-COR, Lincoln, Nebraska, USA). Photosynthesis and respiration measurements were performed ~ 2 weeks after anthesis. The flag leaf photosynthetic assimilation rate (*A*) was estimated at a saturating PPFD of 1500 μmol m^−2^s^−1^ and 30 ºC. Ear photosynthesis was measured using a hand-made chamber connected to the Li-6400XT as described previously for other purposes (Aranjuelo *et al.*, 2009). Ears were enclosed inside the chamber and ingoing air was passed through the chamber at a rate of 1 l min^–1^. The molar fractions of CO_2_ and humidity were measured with the infrared gas analyser of the LI-6400XT. The CO_2_ partial pressure was maintained as constant with the infrared gas analyser-controlled CO_2_ injection system. To ensure steady-state conditions inside the chamber, the system was left to stabilize for a few minutes. An external light source composed of LED lights was placed around the chamber during the measurement providing a PPFD of ~1000 μmol m^–2^ s^–1^ on the ear surface. The photosynthetic rates of the ear presented here are based on the whole organ area. To estimate the ear area, the projected ear surface area of the frontal and the lateral sides were measured with an area meter LI3050A/4 (LI-COR, Lincoln, Nebraska) as has been described before for ear area estimations (Abbate *et al.*, 1997; Shearman *et al.*, 2005). In short, the projected ear surface areas of the frontal and the lateral sides of the ear were measured, and the total value averaged, obtaining similar values to those observed by Teare and Peterson (1971). Dark respiration of the flag leaf and the ear were measured immediately after the photosynthetic measurements at a temperature of 30 ºC. During 2012 growing season the photosynthetic and respiration rates of the flag leaf blade and the ear from genotype ‘PBW343*2/KUKUNA*2//FRTL/PIFED2’ were discarded due to the early phenology of this line.

### Incoming radiation and potential production

Incident and transmitted photosynthetically active radiation (PAR) was measured ~1 week after anthesis on clear days as close to solar noon as possible (11:00–14:00h), with a Linear PAR Ceptometer (AccuPAR LP-80, Decagon, Washington, USA). Different strata of the canopy were considered for the measurements of transmitted PAR: base of the ear (placing the Ceptometer just below the ear) and flag leaf blade (placing the Ceptometer below the flag leaf, which also included the peduncle). The probe of the Ceptometer was held in a representative orientation, diagonally across the two central raised beds with each consisting of two rows of plants. The photosynthetic role of the ear and even of the flag leaf may be underestimated from midday LI measurements because incoming light in the morning and afternoon is not zenithal (i.e. it is oblique). Therefore, the ear in particular (which stands vertically in the top of the canopy), is probably absorbing a larger percentage of incident radiation than inferred from the midday measurements. The light intercepted by each stratum was estimated from the PAR measured by adapting the equations described by Pask *et al.* (2012). The integrated incoming radiation from heading to maturity (MJ_H-M_) was calculated and divided by the number of ears per unit ground area. Incoming radiation was measured with a solar sensor (Eppley PSP at 1000W m^−2^) integrated into a weather station (Davis Wireless Vantage Pro2™ Plus with 24-Hr Fan Aspirated Radiation Shield) located in the CIMMYT experimental station. Thereafter, the (MJ_H-M_) was multiplied by the light interception in the ear and flag leaf strata in order to obtain the integrated incoming radiation in those strata. Furthermore the potential production was calculated from the integrated incoming radiation in the ears and flag leaf assuming a photosynthetic efficiency (solar energy conversion efficiency) of 2.4% (Zhu *et al.*, 2008).

### Inhibition of photosynthesis with DCMU

**Fig. 1.**
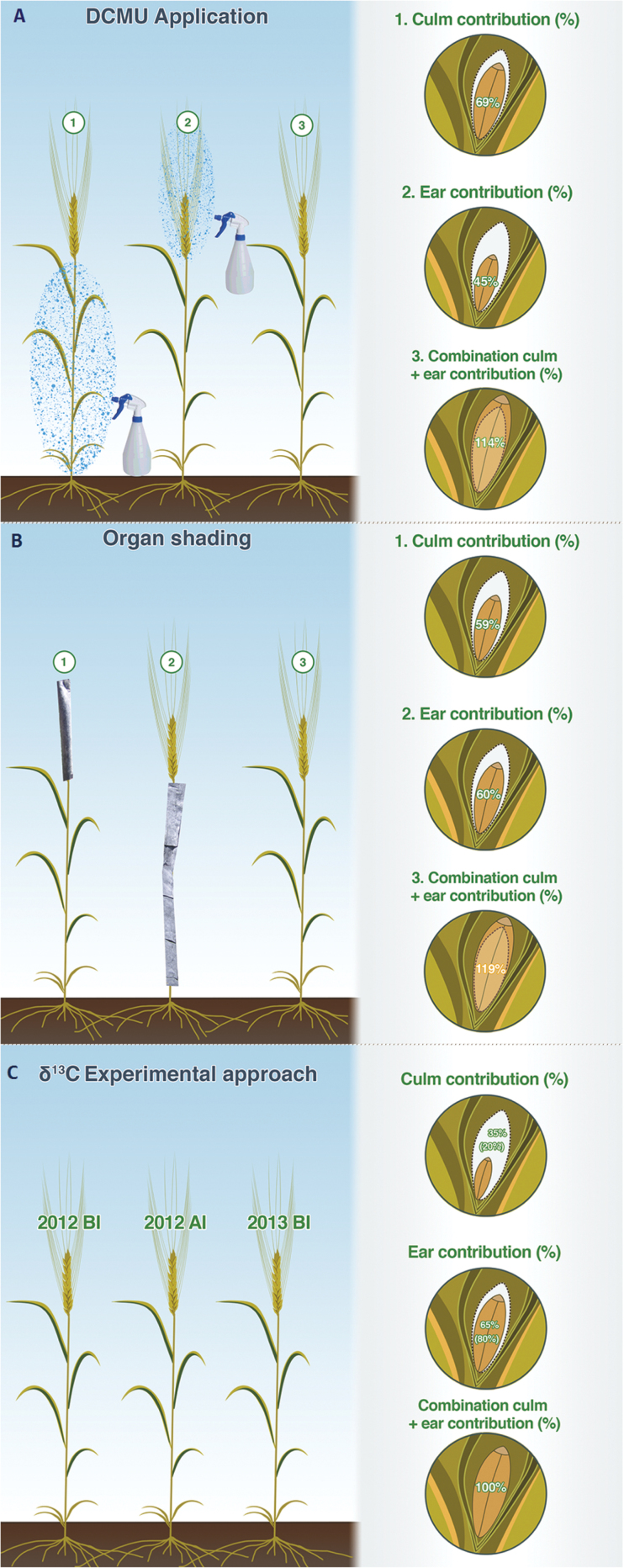
Illustration of a wheat plant showing the relative photosynthetic contributions of the ear and culm to grain filling as estimated with three methodologies: (A) DCMU treatment (2012 growing cycle) with DCMU application to the culm (1) and the ear (2) and a combination of culm plus ear contribution to grain filling (3); (B) shading treatment (2013 growing cycle) with shaded ear (1) and shaded culm (2) and a combination of culm plus ear contribution to grain filling (3); and (C) the δ^13^C approach of the six genotypes of bread wheat. The percentage contribution (%) of the culm and ear were calculated relative to control (see ‘Materials and methods’ equation 1 and Supplementary Table S3). The relative (%) culm and ear contribution to grain filling in the δ^13^C approach was calculated as the proportion of the δ^13^C_peduncle_ and δ^13^C_awns_ contributing to the δ^13^C of mature kernels. Values presented are the averages of values calculated using δ^13^C_peduncle_ and δ^13^C_awns_ values of the WSF from samples taken in the 2012 crop cycle (BI and AI) and for the 2013 crop cycle. The contribution of the ear considering the glumes (in addition of the awns) was also calculated using δ^13^C_peduncle_ and δ^13^C_awns+glumes_ values of the WSF from samples taken in the 2012 BI crop cycle (and for the 2013 crop cycle (value expressed between brackets). For all three approaches values presented are the averaged values ±SD of the six bread wheat genotypes and three replications per genotype. The experiment was performed under field conditions at the CIMMYT’s Experimental Station, Norman E. Borlaug (CENEB), under fully irrigated conditions (This figure is available in colour at *JXB* online.).

Five main tillers were randomly selected in each plot for the photosynthetic inhibition treatments in 2012. Seven days after anthesis, ears or culms (including leaf blades, sheaths and the peduncle) were sprayed with DCMU (3’-(3,4-dichlorophenyl)-1’,1’-dimethylurea) in order to inhibit photosynthesis (Fig. 1A). DCMU is a specific inhibitor of photosynthetic electron transport through photosystem II, which has been observed to be transported by the xylem (Bayer and Yamaguchi, 1965). The inhibition of photosynthesis was checked by measuring photosynthetic gas exchange 3–4 d after DCMU application. Subsequently, the carbon isotopic composition (δ^13^C) in mature grains of different treatments was analysed (see ‘Carbon isotope analysis’ section below).

### Shading treatment

A total set of three main tillers was randomly selected in each plot for the shading treatment in 2013. Eight days after anthesis the ear and the culm (leaf blades, sheaths and the peduncle) were shaded (Fig. 1B). In the shaded culm treatment, the entire vegetative part of the plant was covered, enabling the ears to remain in full sunlight. Shading treatment consisted of wrapping each ear, culm or entire tiller with textile foil, such that light transmitted is below the light compensation point while being gas permeable to avoid ethylene accumulation (Molero *et al.*, 2014).

At maturity, the weight and number of grains per ear in the different treatment (including DCMU and shading) and control groups were measured in order to estimate the photosynthetic contribution of the ear and the culm to grain filling (%). Calculation of the photosynthetic contribution of the ear and the culm to grain filling (organ contribution) was based on the grain weight per ear (GW_ear_) of the treatments relative to the control (Maydup *et al.*, 2010), as follows in equation 1:

$$\text{Organ contribution }\left(\text{\%}\right)=\left[\frac{{\text{GW}}_{\text{ear}}\text{ of treated plants}}{{\text{GW}}_{\text{ear}}\text{ of control plants}}\right]\;\times \;100$$ (1)

where the ear contribution (%) was estimated using the grain weight of the ears with the culm photosynthesis inhibited, whereas the culm contribution (%) was calculated using the grain weight of the ears with the ear photosynthesis inhibited.

### Carbon isotope analysis

Carbon isotope composition was analysed in plants around mid-grain filling. In 2012, samples were collected 17 and 24 d after anthesis (DAA), before irrigation (named as BI) and after irrigation (named as AI), respectively, and in 2013, 18 DAA (Fig. 1C). In the growth chamber (see below), samples were collected 8 weeks after sowing. For each sampling in the field, ten representative ears, flag leaves, and peduncles per plot were harvested. In 2012, a full set of BI samples were collected and immediately frozen with liquid nitrogen. For AI samples collected in 2012, only half of the samples were frozen with liquid nitrogen immediately after sampling (named ‘frozen’). The other half were stored at room temperature in paper bags for~3h after sampling (named ‘not-frozen’). All samples from the 2012 trial (samples frozen with liquid nitrogen and samples stored in paper bags) were finally stored at −20 ºC and then lyophilized for 48h. For the 2013 season, samples were stored at room temperature for ~3h after sampling and subsequently oven dried at 70 ºC for 48h. Once dried, the glumes, awns, flag leaves, and peduncles were separated, weighed, and finely ground.

The stable carbon isotope composition (δ^13^C) in the water-soluble fraction (WSF) of the peduncles, awns, glumes, and leaves in the field trials (and only leaves in the growing chamber experiment) were analysed as described previously (Yousfi *et al.*, 2013). Glumes were only analysed in 2012 BI and in 2013. The δ^13^C was also analysed in mature kernels. Approximately 1mg of each dry sample (100 µl for WSF) was weighed into tin capsules and measured with an elemental analyser coupled with an isotope ratio mass spectrometer (Delta C IRMS, ThermoFinnigan, Bremen, Germany) operating in continuous flow mode in order to determine the stable carbon (^13^C/^12^C) isotope ratios of the same samples as explained elsewhere (Sanchez-Bragado *et al.*, 2014a, *b*). Isotopic analyses were carried out in the Scientific-Technical Services of the University of Barcelona, Spain.

### Relative photosynthetic contribution to grain filling

The approach proposed here considers that the relative contribution of the awns and peduncle to grain filling varies as a result of water status and that it is reflected in the δ^13^C of mature grains (Araus *et al.*, 2003). Based on the approach developed and explained in detail previously (Sanchez-Bragado *et al.*, 2014b) it is expected that the δ^13^C of the kernels (δ^13^C_grain_) will directly reflect the isotopic signal resulting from the combinations of the δ^13^C of assimilates coming from different photosynthetic sources. This implies that the same slope and origin at zero needed to be found between the combined δ^13^C of the peduncle (δ^13^C_peduncle_) and the awns (δ^13^C_awns_) and the δ^13^C of the kernels. The approach was performed during the 2012 (BI and AI) and 2013 crop seasons. In addition, the approach was also performed considering both the awns and the glumes as the photosynthetic organs of the ear. Thus, the δ^13^C of the awns plus the glumes (δ^13^C_awns+glumes_) and the peduncle were compared with the δ^13^C of mature kernels using a linear fit. The adjusted δ^13^C values of the awns plus the glumes were obtained by multiplying each organ by the pondered average dry weight of each organ in order to take into account the relative dimension of each tissue.

### Effect of sampling conditions on δ^13^C of the water-soluble fraction

In order to reduce possible divergences in the δ^13^C of the water-soluble fraction triggered by the different sampling and drying methods used during 2012 (BI and AI) and between the 2012 and 2013 seasons, a correction factor (CF) was calculated (Supplementary Table S1) according to equation 2:

CF=[AI not frozen−BI frozen]−[AI frozen−BI frozen] (2)

The input parameters used for calculating the correction factor were the δ^13^C in the WSF of the peduncle, awns, and flag leaves collected in 2012. The correction factor was estimated to be on average 0.4 ‰ (Supplementary Table S1). In 2012 samples collected AI (not frozen) were corrected with the CF obtained in equation 2. This CF was applied to the δ^13^C in the WSF by adding a constant CF of 0.4‰ to each individual value.

### Experimental estimation of the effect of sampling conditions on δ^13^C in the WSF

A modern Spanish durum wheat (*Triticum turgidum* L. var. *durum*) cultivar (Regallo) was grown in 3 l pots (three replicates) filled with sand (one plant per pot). Plants were watered three times a week with Hoagland nutrient solution and were grown under controlled conditions in a growth chamber (Conviron E15, Controlled Environments Ltd, Winnipeg, Canada). Plants were supplied with a PPFD of ~400 µmol m^−2^ s^−1^ at plant level during the light period (14h). A constant relative humidity of 50–60% and a temperature of 23/17 ºC during the light and dark periods, respectively, were also maintained. Three leaves of each plant were collected and divided longitudinally into two parts. One segment was frozen with liquid nitrogen immediately after sampling and the other segment (of the same leaf) was oven-dried 6h after sampling for 48h. Further leaf segments were finely ground. Subsequently, δ^13^C in the WSF of leaf segments was analysed as previously mentioned (see the ‘Carbon isotope analysis’ section above).

In order to confirm the existence of possible discrepancies in the δ^13^C of the water-soluble fraction triggered by different sampling and drying methods, an additional correction factor was experimentally calculated in leaves obtained in the growing chamber experiment (Supplementary Table S2). The correction factor was calculated from the difference in δ^13^C in the WSF between leaves oven-dried 6h after sampling and leaves frozen with liquid nitrogen and subsequently lyophilized. The correction factor was estimated to be on average 0.8 ‰ (Supplementary Table S2). Samples collected in 2013 (which were oven-dried) were corrected with the CF obtained in the growth chamber experiment. This CF was applied to the δ^13^C in the WSF by adding a constant CF of 0.8‰ to each individual value.

### Statistical analysis

One-way analysis of variance (ANOVA) using the general linear model was calculated in order to quantify the effects of genotype and organ interaction on the studied parameters. Genotype and organ were included as fixed factors including three blocks and three replicates per block. Means were compared by Tukey’s honestly significant difference test. A bivariate correlation procedure was constructed to analyse the relationships between the measured traits. Statistical analyses were performed using the SPSS 21.0 statistical package (SPSS Inc., Chicago, IL, USA). Figures were created using the Sigma-Plot 10.0 program (SPSS Inc.).

## Results

### Contribution of the ear and the culm to grain filling: DCMU application

In order to estimate the relative contribution of the ear and the culm to filling grains, the photosynthesis of either ears or culms (which represent all the assimilation organs below the ear) was inhibited with DCMU (Table 1). Mean values of carbon isotope composition in mature grains of control plants (δ^13^C_grain_) were higher (less negative δ^13^C) in comparison to the δ^13^C_grain_ in DCMU-ear plants (ear photosynthesis inhibited) but similar to the δ^13^C_grain_ in DCMU-culm plants (culm photosynthesis inhibited); however, grain weight per ear (GW_ear_) and thousand kernel weight (TKW) in the DCMU-culm plants showed the lowest values (19.8g and 1.29g, respectively) compared to the control plants (44.3g and 2.89g, respectively) and the DCMU-ear treatment (31.6g and 1.99g, respectively), whereas the number of grains per ear (NG_ear_) did not differ within the treatments and control plants. In addition, genotypic differences existed for δ^13^C_grain_ and GW_ear_ and TGW, whereas genotype comparisons to treatment interactions were not significant.

**Table 1.** Mean values of stable carbon isotope composition in mature grains (δ^13^C_grain_), total grain weight per ear (GW_ear_), the number of grains per ear (NG_ear_) and thousand kernel weight (TKW) in control, DCMU-culm (inhibition of the whole culm photosynthesis) and DCMU-ear (inhibition of ear photosynthesis) plants Analysis of variance (ANOVA) for the effect of genotype and treatment is shown. Mean values with different superscripted letters are significantly different according to the Tukey’s honestly significant difference test (*P*<0.05). Each value represents five genotypes and three replications per genotype (one genotype was discarded due to early phenology). Experiment performed in the 2012 crop season.

**Treatment**	**δ** ^**13**^ **C**_**grain**_ **(‰)**	**NG**_**ear**_	**GW**_**ear**_ **(g)**	**TKW (g**)
DCMU culm	−26.0^b^	65.7^a^	1.29^a^	19.8^a^
DCMU ear	−26.7^a^	62.7^a^	1.99^b^	31.6^b^
control	−26.3^b^	65.5^a^	2.89^c^	44.3^c^
**Level of significance**				
Genotype (G)	0.000^***^	ns	0.000^***^	0.009^***^
Treatment (T)	0.008^**^	0.000^***^	0.009^**^	0.000^***^
G×T	ns	ns	ns	ns

### Monitoring effects of DCMU on photosynthesis

In order to monitor the efficiency of the inhibition method with DCMU, the photosynthesis of the ear and the flag leaf blade (Table 2) was measured. As expected, when DCMU was applied to ears, the net ear photosynthesis was significantly inhibited (−11.45 μmol·m^−2^·s^−1^) compared to the control ears (9.95 μmol·m^−2^·s^−1^). Concerning the flag leaf blade, net photosynthesis was not inhibited when DCMU was applied to the ears. Thus, net photosynthetic rates in the flag leaf blade showed similar values to the DCMU ear treatment (17.95 μmol·m^−2^·s^−1^) and control (18.66 μmol·m^−2^·s^−1^); however, when DCMU was applied to the culms, net photosynthesis was not only inhibited in the stem, but also the photosynthesis of the ears was affected (3.82 μmol·m^−2^·s^−1^).

**Table 2.** Mean values of ear and flag leaf blade photosynthesis expressed as the instantaneous net photosynthetic rate and instantaneous dark respiration for the control and the two DCMU treatments Analysis of variance (ANOVA) for the effect of genotype and treatment is shown. Mean values with different superscripted letters are significantly different according to the Tukey’s honestly significant difference test (*P*<0.05). Each value represents five genotypes and three replications per genotype (one genotype was discarded due to early phenology). Experiment performed in the 2012 crop season.

	**Flag leaf (μmol·m** ^**−2**^ **·s** ^**−1**^)		**Ear (μmol·m** ^**−2**^ **·s** ^**−1**^)	
	**Net photo.**	**Dark resp.**	**Net photo.**	**Dark resp.**
DCMU culm	−1.804^a^	−1.945^b^	3.823^b^	−16.690^a^
DCMU ear	18.662^b^	−3.049^ab^	−11.446^a^	−13.895^a^
Control	17.950^b^	−3.697^a^	9.947^c^	−17.170^a^
**Level of significance**				
Genotype (G)	ns	ns	0.028^**^	ns
Treatment (T)	0.000^***^	0.018^*^	0.000^***^	ns
GxT	ns	ns	Ns	ns

### Contribution of the ear and the culm to grain filling: shading treatment

Mean values of NG_ear_ and TKW for shaded-culms and shaded-ears were both lower than control plants (Table 3). Moreover, mean values of GW_ear_ were similarly affected by shading the ears and the culms, and did not show significant differences. Both NG_ear_ and GW_ear_ exhibited genotypic effects, whereas only GW_ear_ showed significant genotype × environment interactions.

**Table 3.** Mean values in the set of six selected genotypes of total grain weight per ear (GW_ear_), the number of grains per ear (NG_ear_) and thousand kernel weight (TKW) in control, shaded-ear and shaded-culm plants Analysis of variance (ANOVA) for the effect of genotype and treatment is shown. Mean values with different superscripted letters are significantly different according to the Tukey’s honestly significant difference test (*P*<0.05). Experiment performed in the 2013 crop season.

**Treatment**	**NG**_**ear**_	**GW**_**ear**_ **(g)**	**TKW (g**)
Shaded ear	50.8^a^	1.50^a^	30.2^b^
Shaded culm	55.3^b^	1.51^a^	27.3^a^
Control	60.9^c^	2.53^b^	41.5^c^
**Level of significance**			
Genotype (G)	0.000^***^	0.000^***^	0.000^***^
Treatment (T)	0.000^***^	0.000^***^	0.000^***^
G×T	ns	0.003^*^	ns

### Monitoring effects of shading treatments on photosynthesis

In order to monitor the reliability of the shading method, the photosynthesis of the ear and the flag leaf blade (Table 4) was measured. Mean values of flag leaf blade photosynthesis under shaded ear treatment (16.15 μmol·m^−2^·s^−1^) were not significantly different compared to control (17.89 μmol·m^−2^·s^−1^). Whereas ear photosynthesis under shaded culm treatment (8.64 μmol·m^−2^·s^−1^) was higher compared to control (6.44 μmol·m^−2^·s^−1^), dark respiration was lower under shaded culm treatment (−9.67 μmol·m^−2^·s^−1^) compared to control (−13.24 μmol·m^−2^·s^−1^). In addition, dark respiration in the ear was higher in control than shaded-culm plants (Table 4).

**Table 4.** Mean values of ear photosynthesis and flag leaf blade expressed as instantaneous net photosynthetic rate and instantaneous dark respiration for control, shaded ear and shaded culm treatments Analysis of variance (ANOVA) for the effect of genotype and treatment is shown. Mean values with different superscripted letters are significantly different according to the Tukey’s honestly significant difference test (*P*<0.05). Each value represents six genotypes and three replications per genotype. Experiment performed in the 2013 crop season.

	**Flag leaf (μmol·m** ^**−2**^ **·s** ^**−1**^)		**Ear (μmol·m** ^**−2**^ **·s** ^**−1**^)	
	**Net photo.**	**Dark** **respiration**	**Net photo.**	**Dark** **respiration**
Shaded ear	16.145^a^	−0.982^a^	-	-
Shaded culm	-	-	8.635^b^	−9.671^b^
Control	17.892^a^	−0.968^a^	6.438^a^	−13.236^a^
**Level of significance**				
Genotype (G)	ns	ns	0.016^*^	ns
Treatment (T)	ns	ns	0.012^*^	0.000^***^
G×T	ns	ns	ns	ns

### Monitoring sampling procedure

In order to monitor the outcome of the sampling procedure (samples frozen or not frozen), mean values of δ^13^C in the WSF of the peduncle and awns minus the mean values of δ^13^C in the mature kernels (δ^13^CWSF_P_ − δ^13^C_G_ and δ^13^CWSF_A_ − δ^13^C_G_, respectively) were compared in 2012 (Supplementary Table S1). Differences within the δ^13^C in the WSF of the peduncles and awns when compared to the grains were on average higher in the plots in which plants were not frozen immediately (−2.03‰) relative to frozen plants (−1.61‰). In addition, differences within the peduncles and awns sampled BI and AI in 2012 (δ^13^C WSF_AI-P_ − δ^13^C WSF_BI-P_, and δ^13^C WSF_AI-A_ − δ^13^C WSF_BI-A_, respectively), were calculated for frozen and not-frozen samples (Supplementary Table S1). Thus, samples from plots that were not frozen exhibited greater differences within organs sampled BI and AI (−0.91‰) compared to the plots whose plants were frozen (0.51‰).

### Photosynthetic contribution of the ear and the culm to grain filling: δ^13^C comparison

**Fig. 2.**
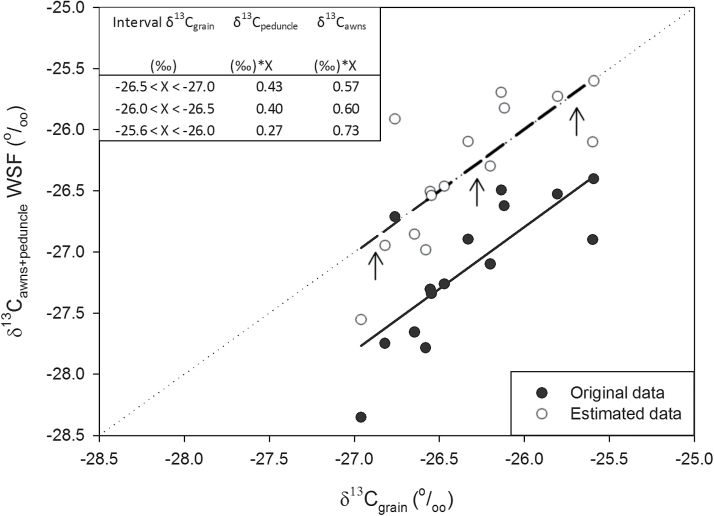
Linear regression of the relationship between the stable carbon isotope composition in mature grains (δ^13^C_grain_) and the combination of δ^13^C from awns and the peduncle (δ^13^C_awns_+δ^13^C_peduncle_) in the water-soluble fraction (WSF) during the 2013 crop season (R^2^=0.58; *P*=0.001). Closed symbols (original data) indicate raw data, and open symbols (estimated data) indicate original data with a correction factor of 0.8 ‰ (see ‘Materials and methods’ and Supplementary Table S2). The six genotypes and three replications per genotype were considered, accounting for a total of 18 plots. For each plot the relative weight assigned to the δ^13^C of each of the two organs depended on the water status of the plot assessed by its δ^13^C_grains_ (see figure inset). The experiment was performed under field conditions at the CIMMYT’s Experimental Station, Norman E. Borlaug (CENEB), under fully irrigated conditions.

The relative contribution of the δ^13^C_awns_ and the δ^13^C_peduncle_ that accounted for the δ^13^C_grains_ was assessed through a linear fit (Table 5, Fig. 2). The δ^13^C_grain_ was used as a dependent variable and the δ^13^C in the WSF of awns and peduncles were used as the independent variables, with assignment of a different weight for the δ^13^C of the awns and peduncles depending on the δ^13^C_grain_. Thus, in 2012 before irrigation (Table 5) the δ^13^C_awns_ showed a relative contribution of 75% (δ^13^C_awns_×0.75) and the peduncles 25% (δ^13^C_peduncle_×0.25), when the δ^13^C_grain_ values were between −25.2 ‰ and −25.8‰. Conversely, the relative contribution of the awns was 25% (δ^13^C_awns_×0.25) and the peduncle 75% (δ^13^C_peduncle_×0.75) when δ^13^C_grain_ values were between −26.4‰ and −27.0‰. In this way a linear fit with a slope of one and origin at zero was achieved (R^2^=0.61, *P*<0.001). Furthermore, the awns showed a higher relative contribution in the linear regression in 2012 after irrigation (Table 5) compared to linear regression before irrigation. As mentioned in the ‘Materials and methods’ section, values in the δ^13^C in the WSF of awns and peduncle (not frozen samples obtained AI in 2012) were re-calculated using the experimentally calculated correction factor (Supplementary Tables S1, S2). Hence, from the linear fit after irrigation (R^2^=0.70 *P*<0.001), the relative contribution of the δ^13^C_awns_ ranged from 66% (when δ^13^C_grain_ values were within the most negative interval, −26.4‰ and −27.0‰) to 100% (when the δ^13^C_grain_ values were within the most positive interval, −25.2 ‰ and −25.8‰).

**Table 5.** Pearson correlation coefficient of the relationship between stable carbon isotope composition in mature grains (δ^13^C_grains_) and the combination of the δ^13^C from the peduncle and the awns (δ^13^C_peduncle_+δ^13^C_awns_) in the water-soluble fraction (WSF) ‘Peduncle (%)’ represents the relative contribution of the culm (i.e. the whole plant below the ear) to grain filling as a percentage and ‘awns (%)’ represents the relative contribution of the awns to grain filling as a percentage. The individual values of δ^13^C_awn_ and δ^13^C_peduncle_ used in the linear regression belong to δ^13^C in the WSF before and after irrigation during the 2012 crop season. After irrigation for samples that were not frozen with liquid nitrogen a correction factor of 0.4‰ in the values of the δ^13^C in the WSF was applied (see ‘Materials and methods’ and Supplementary Table S1). The six genotypes and three replications per genotype were considered, accounting for a total of 18 plots per sampling date. For each plot the relative weight assigned to the δ^13^C of each of the two organs depended on the water status of the plot assessed by its δ^13^C_grains_ based on Sanchez-Bragado *et al.* (2014b). Level of significance: ***, *P*<0.001.

	**Interval δ** ^**13**^ **C**_**grain**_ **(‰)**		**Awns (%**)		**Peduncle (%**)	R^2^
**Before irrigation**						
	[−25.2, −25.8]		75		25	
	[−25.8, −26.4]		50		50	
	[−26.4, −27.0]		25		75	
	δ^13^C_grain_	*vs*	[δ^13^C_awns_*(%)	+	δ^13^C_peduncle_*(%)]	0.61^***^
**After irrigation**						
	[−25.2, −25.8]		100		0	
	[−25.8, −26.4]		80		20	
	[−26.4, −27.0]		66		33	
	δ^13^C_grain_	*vs*	[δ^13^C_awns_*(%)	+	δ^13^C_peduncle_*(%)]	0.70^***^

**Fig. 3.**
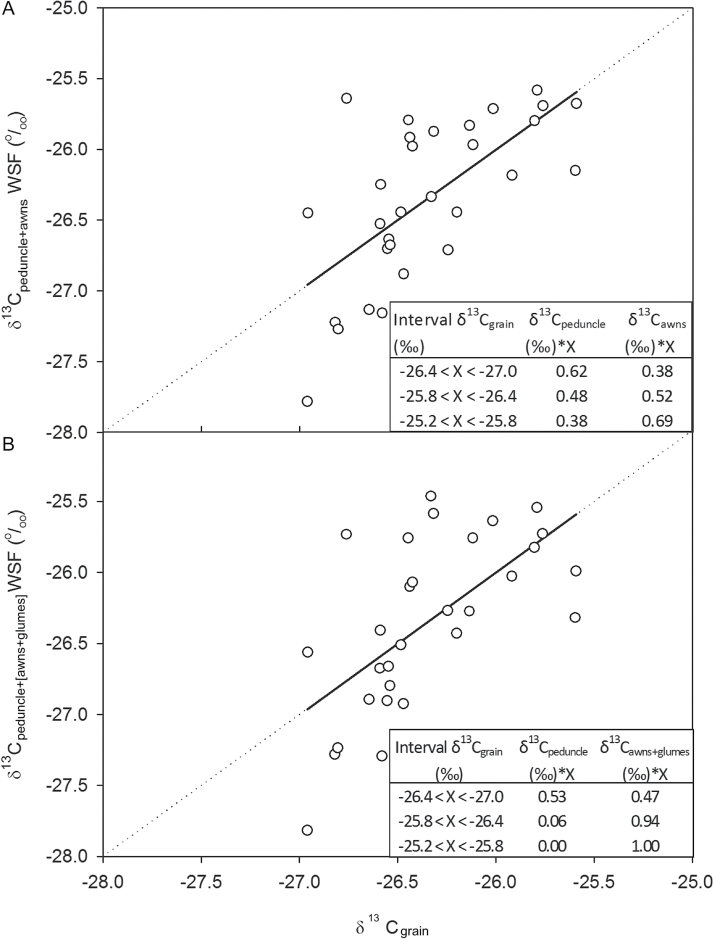
Linear regression of the relationship between the stable carbon isotope composition in mature grains (δ^13^C_grain_) and (A) the combination of δ^13^C from awns and the peduncle (δ^13^C_peduncle+awns_) in the water-soluble fraction (WSF) (R^2^=0.44, *P*<0.001) and (B) the combination of δ^13^C from awns and glumes and the peduncle (δ^13^C_peduncle+[awns+glumes]_) in the water-soluble fraction (WSF) (R^2^=0.40, *P*<0.001) during the 2012 and 2013 crop seasons. For the 2012 season only data before irrigation were used. In 2013 a CF of 0.8 ‰ was applied. Other details as in Fig. 2. The experiment was performed under field conditions at the CIMMYT’s Experimental Station, Norman E. Borlaug (CENEB), under fully irrigated conditions.

Conversely, in 2013 the relative contribution of the δ^13^C_awns_ and the δ^13^C_peduncle_ that accounted for the δ^13^C_grain_ was achieved through a linear fit (R^2^=0.58; *P*=0.001) with a slope of one but without an origin at zero (Fig. 2). Thus, the δ^13^C in the WSF of awns and peduncles exhibited more negative values in 2013 compared to the 2012 experiment. As mentioned in the ‘Materials and methods’, values of δ^13^C in the WSF of awns and peduncles obtained in 2013 were recalculated with the correction factor (Supplementary Table S2) to account for the deviation associated with sampling and further drying conditions, whereby a linear fit with a slope of one and an origin at zero was then possible to achieve. Hence, the relative contribution of the δ^13^C_awns_ in the linear fit (Fig. 2) was quite steady, ~63% irrespective of the δ^13^C_grain_ values. Moreover, in order to account for the photosynthetic contribution of the glumes to grain filling, the same approach as in Fig. 2 was performed but this time it also considered the δ^13^C in the WSF of the glumes. To that end we used the samples of the two seasons (samples collected in 2012 before irrigation and in 2013) where the δ^13^C of the WSF of the glumes were analysed. We compared the results with and without the inclusion of the glumes. In the first case the linear regression was performed combining the δ^13^C from the awns and the peduncle (δ^13^C_peduncle+awns_) in the WSF (Fig. 3A), whereas in the second scenario the combination of δ^13^C from awns and glumes against the peduncle [δ^13^C_peduncle+(awns+glumes)_] in the WSF was compared (Fig. 3B). Hence, the relative contribution of the awns and glumes to grain filling (δ^13^C_awns+glumes_) was higher (on average 80%) compared to relative contribution when only the awns (δ^13^C_awns_) were considered (on average 53%) in the linear fit.

Summarizing, the DCMU approach assigned a lower relative contribution, with the mean value ±SD being 45.4±6.0% for the ear compared to the organ shading and the δ^13^C approaches (between 60% and 65%, respectively). Besides, compensatory effects were observed in the DCMU and shading treatment. Hence, the relative contribution of ears and culm together (Fig. 1, Supplementary Table S3) accounted for more than the expected for the intact (100%) plants, mean values ±SD being 114±16% for DCMU and 120±12% for shading treatment. Concerning the δ^13^C in the WSF, the greatest relative contribution was observed in the ear (65±21%) (not considering the glumes) compared to DCMU and shading treatments.

### Potential biomass production

**Fig. 4.**
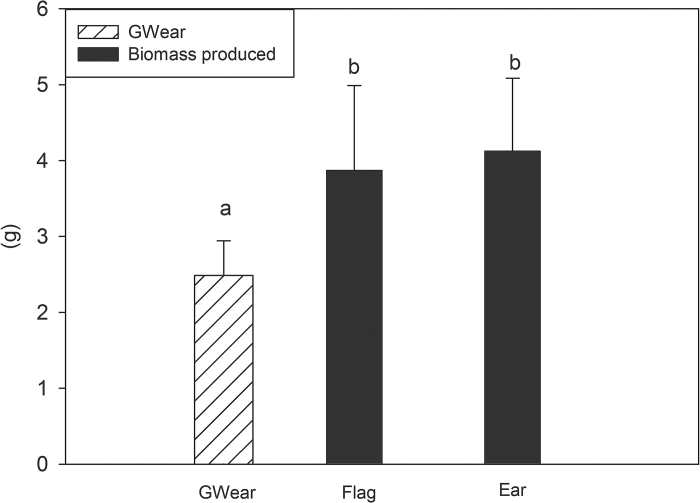
Comparison of kernel weight per ear (g) at maturity (GW_ear_) with the photosynthetic contribution of the ear and the flag leaf during grain filling, estimated from the potential biomass (g) produced by each of the two organs from heading to maturity based in the time-integration of the irradiance intercepted by the canopy layers where the ear and the flag leaf are placed (see ‘Material and methods’ section). Each bar represents the mean values ±SD of the six genotypes and the three replications per genotype during the 2012 and 2013 crop cycle (one genotype was discarded due to early phenology in 2012). Mean values with different superscripted letters are significantly different according to the Tukey’s honestly significant difference test (*P*<0.05). The experiment was performed under field conditions as described in previous figures.

The potential amount of biomass produced by the flag and the ear (Fig. 4), as inferred from light interception and photosynthetic assimilation accumulated from heading to maturity (see ‘Materials and methods’), surpassed the total grain weight of the ear.

## Discussion

### Photosynthetic contribution of the ear and the culm to grain filling: DCMU application

The GW_ear_ exhibited lower values with DCMU applied to the culm compared to GW_ear_ with DCMU applied to the ear (Table 1). This indicates that the organ that most affected grain filling following photosynthesis inhibition was the culm. Thus, the culm contributed around 69±4%, whereas the ear contributed 45±6% (Fig. 1, Supplementary Table S3). However, when the DCMU was applied to the culm, not only was the photosynthesis of the stem (measured in the flag leaf) affected (Table 2), but the photosynthesis of the ear was also partly inhibited. These results suggest that DCMU is transported acropetally to the ear, causing a premature yellowing of awns and glumes, but that DCMU is not transported to the culm from the ears. In fact, it has been observed in red kidney bean, soybean, and barley that diuron (where the active ingredient is DCMU) moves in the direction of the transpiration stream but not basipetally, unlike the assimilates moving from the leaves to the lower parts of the plant (Bayer and Yamaguchi, 1965). In contrast, some evidence of movement of 2,4-dichloro-phenoxyacetic acid (2,4 D) has not been only observed in the transpiration stream (xylem) at low pH in barley (Shone and Wood, 1974; Shone *et al.*, 1974) but also in the phloem (Craft and Yamaguchi, 1958). Such findings suggest that ‘desiccants’ were transported from stem and leaves (via phloem) to the ears and subsequently to the growing grains (Blum *et al.*, 1983). In similar studies leaves and stems sprayed with potassium iodide resulted in a lower reduction in grain growth (Herrett *et al.*, 1962; Saeidi *et al.*, 2012) compared to treatments with other desiccants such as monuron (3’-(4-chlorophenyl)-1,1-dimethylurea) (alias CMU) which is primarily an inhibitor of PSII, although it is an order of magnitude less effective and can be transported through the phloem (Yamaguchi and Crafts, 1959).

### Photosynthetic contribution of the ear and the culm to grain filling: shading treatment

The importance of ear photosynthesis was also supported by the other two experimental approaches of this study. In the textile-shading approach, mean values of GW_ear_ from shaded ears (Table 3) were similar to shaded culms, indicating that ear photosynthesis was similar to culm photosynthesis (leaf blades, sheaths and peduncles) in terms of contribution to grain filling; however, the intrusive nature of treatments such as DCMU or shading should be kept in mind. These results should therefore be interpreted with caution because potential compensation effects triggered by these treatments may eventually increase the contribution of unaffected photosynthetic organs or preanthesis reserves to grain filling (Aggarwal *et al.*, 1990; Eyles *et al.*, 2013). Indeed, the total contribution to grain filling attributed to the ear and culm together in DCMU (114±16%) and shading (120±12%) treatments was higher than the control (100%), suggesting possible compensation effects by unaffected photosynthetic organs (Fig. 1, Supplementary Table S3) as has been previously reported (Aggarwal *et al.*, 1990; Chanishvili *et al.*, 2005; Ahmadi *et al.*, 2009).

### Photosynthetic contribution of the ear and the culm to grain filling: δ^13^C comparison

In a less invasive manner, the δ^13^C approach aims to assess the relative contribution of different photosynthetic organs that are active in providing assimilates to the grains during grain filling. The δ^13^C approach avoids the unwanted compensatory mechanisms and chemical effect of current methods derived from a plant part-specific photosynthesis limitation. Bearing this in mind, the δ^13^C approach showed on average a higher relative contribution from the awns compared to the peduncles (Table 5, Fig. 2), highlighting the relative importance of ear photosynthesis compared to green culm parts (the peduncle integrates leaf blades and sheaths). In addition the relative contribution to grain filling of the awns plus the glumes (δ^13^C_awns+glumes_) was higher (80% on average) compared to the relative contribution when only the awns (δ^13^C_awns_) were considered (53% on average) (Fig. 3). Awns, when present, are considered the main photosynthetic tissues of the ear fixing external (atmospheric) CO_2_ (Li *et al.*, 2006; Tambussi *et al.*, 2007*b*). In fact, the net photosynthetic rates of awned bread wheat lines have been observed as being two to three times greater than those of their isogenic awnless counterparts (Olugbemi *et al.*, 1976).

The δ^13^C approach (using δ^13^C in the WSF) may help to estimate the proportion of assimilates produced by the awns and peduncles that are ready to be transported (Brandes *et al.*, 2007) under short-term environmental conditions. However, some assumptions were considered in the isotopic approach, such as secondary fractionation during storage, and mobilization to the grains is minimal (Cernusak *et al.*, 2009). Regarding this point, Raven and Griffiths (2015) argued that the ^13^C enrichment that we observed in the peduncle could be the consequence of secondary fractionation during (re)mobilization and storage of carbohydrates (e.g. in the leaf flag) instead of constitutive differences in the δ^13^C associated with the peduncle (Hubick and Farquhar, 1989; Araus *et al.*, 1993). Although fractionation of carbohydrates due to remobilization to the grains has not been demonstrated in wheat (Yoneyama *et al.*, 1997), a possible displacement of stored carbohydrates at night might occur (Tcherkez *et al.*, 2004). This point should be taken into account as we only sampled during the day. However, studies in sunflower and wheat could not demonstrate a clear daily variation in δ^13^C carbohydrates (Ghashghaie *et al.*, 2001; Kodama *et al.*, 2011). Moreover, Raven and Griffiths (2015) also mentioned that organic matter entering the ear via the xylem could be biasing the δ^13^C of the ear. The xylem transports organic acids and amino acids synthesized in roots (C_3_ plants), and these may show a much lower δ^13^C than those compounds synthesized in the leaves (Yoneyama *et al.*, 1997). However, the supply of C via xylem to the ear has been observed as being quantitatively very low compared to phloem (Taiz and Zeiger, 2002).

### Effect of sampling conditions on the δ^13^C of the water-soluble fraction

Although the photosynthetic contribution of the awns to grain filling has been observed to be higher under drought stress conditions (Motzo and Giunta, 2002), our results from 2012 showed on average a higher relative contribution from the awns (Table 5) after irrigation (82%) compared to before irrigation (50%). The increase in the relative contribution of awns after irrigation may have been also related to the sampling method (samples taken prior to irrigation were immediately frozen and part of samples taken after irrigation were not frozen). Greater differences between the δ^13^C in the WSF of the peduncle and the awns versus the δ^13^C of mature kernels in not-frozen samples (Supplementary Table S1) suggest that the δ^13^C in the WSF could have been biased due to the sampling method. Besides, in the growth chamber experiment (Supplementary Table S2), oven-dried leaves showed more negative δ^13^C in the WSF (−32.5‰) compared to leaves frozen with liquid nitrogen (−31.6‰). Therefore, oven-dried samples (in 2013) and samples that were not frozen immediately after sampling (AI 2012) shared a common denominator: metabolic activity was not stopped straight away. Thus, the fact that the metabolic activity after sampling was not halted could have led to organ respiration continuing for a while after sampling (Ocheltree and Marshall, 2004; Gessler *et al.*, 2009*b*). Numerous studies have shown significant ^13^C enrichment of respired CO_2_ compared to the remaining substrate in leaves (Ghashghaie *et al.*, 2001; Gessler *et al.*, 2009*a*), shoots, roots (Kodama *et al.*, 2011), peduncles and awns (Sanchez-Bragado *et al.*, 2014*b*). In this sense, respiration has been observed to rely on current photo-assimilation rather than mobilized reserves (Bell and Incoll, 1990), leading to more negative δ^13^C in the remaining WSF after dark respiration.

### Indirect non-intrusive approaches supporting the key photosynthetic role of the spike

In wheat, not only are the awns important tissues for assimilating atmospheric CO_2_ in the ear (Li *et al.*, 2006), but also the glumes (and other bracts) may be involved in atmospheric CO_2_ fixation in addition to re-assimilating respired CO_2_ (Bort *et al.*, 1996; Maydup *et al.*, 2014). In fact, Fig. 3 supports such findings, where ear contribution increased on average 27% when glumes were considered. However, it appears that, when present, awns are the main photosynthetic organs of the ear (Tambussi *et al.*, 2007*a*) that fix atmospheric CO_2_ (Blum, 1985). Moreover, the potential photosynthetic contribution of the ear to grain filling is also evidenced at the canopy level. The upper part of the canopy (basically constituted by the ears) integrated from heading to maturity and assuming a photosynthetic efficiency of 2.4% (Zhu *et al.*, 2008) represented a potential production of biomass of 4.1 g·per ear. Such potential production was found to be within the range of total grain weight per ear (Fig. 4), providing an indirect support in favour of the ear as the main photosynthetic organ during grain filling under good agronomic conditions. In fact, the potential amount of biomass in the flag leaf and the ear surpassed the GW_ear_ suggesting that during the night a proportion of the assimilates may be respired, leading to a reduction in the GW_ear_. However, during day the glumes may help to re-fix this night-respired CO_2_, indicating the existence, to some extent, of CAM metabolism (Tambussi *et al.*, 2005) in the ear, supporting the already mentioned important role of the ear contribution to grain filling. Moreover, compared to other organs (e.g. the flag leaf), the ear has high respiratory rates (Knoppik *et al.*, 1986; Araus *et al.*, 1993), particularly during mid grain-filling period (Caley *et al.*, 1990; Araus *et al.*, 1993; Bort *et al.*, 1996; Tambussi *et al.*, 2005; 2007*b*), suggesting that growing grains (together with maintenance respiration in the bracts) are actively contributing to dark respiration (Knoppik *et al.*, 1986). In fact, when expressed on a dry matter basis, ear dark respiration values were still 50–60% (data not shown) of those of the flag leaf in spite of the high portion of inert (i.e. support) tissues (mostly of schlerenchymatous nature) in different parts of the ear, such as the bracts and the rachis (Blum, 1985; Araus *et al.*, 1993; Li *et al.*, 2006).

## Conclusions

As far as we know, this is the first report where different, independent experimental approaches of an intrusive and non-intrusive nature were used to assess the contribution of ear photosynthesis to grain filling. The shading approach assigned a similar contribution to the ear as to the culm. The DCMU approach assigned a greater role to the culm but herbicide application to the culm affected the ear, thus biasing the final grain weight. Moreover DCMU and shading approaches may cause compensatory effects which overestimated the contribution of unaffected organs. The δ^13^C approach assigned a higher photosynthetic contribution to the ear than to the culm. Other indirect, albeit non-intrusive approaches of absorbed integrated irradiance also support the role of the ear as a main contributor to filling grains. Moreover, genetic variability was observed with regards to the relative contribution of the ear to grain filling using the δ^13^C approach. However, some consideration should be given when applying the δ^13^C approach, including the sampling method used, in order to take into account post-harvest respiration. Moreover, further research is needed to clarify under which particular conditions ear photosynthesis is a positive trait for improving grain yield.

## Supplementary data

Supplementary data are available at *JXB* online.

Figure S1. Environmental conditions during the growing seasons 2012 and 2013.

Table S1. Mean values of δ^13^C in the WSF of the peduncle, mature kernels, awns and flag leaf minus mature kernels after irrigation and differences in the δ^13^C in the WSF after irrigation minus before irrigation during the 2012 crop season.

Table S2. Values of δ^13^C in the WSF of wheat leaves in the growth chamber experiment.

Table S3. Relative photosynthetic contributions of the ear and culm to grain filling assessed through the three methodologies considered in this study.

Supplementary Data
