# Supplementary material for: Photosynthetic contribution of the ear to grain filling in wheat: a comparison of different methodologies for evaluation
Source: J Exp Bot. 2016 Mar 24;67(9):2787–98. doi: 10.1093/jxb/erw116 (PMC4861024; doi:10.1093/jxb/erw116)
Supplement: Supplementary Data [file supp_67_9_2787__index.html]

Photosynthetic contribution of the ear to grain filling in wheat: a comparison of different methodologies for evaluation — Photosynthetic contribution of the ear to grain filling in wheat: a comparison of different methodologies for evaluation — Supplementary Data 

# Photosynthetic contribution of the ear to grain filling in wheat: a comparison of different methodologies for evaluation

## Supplementary Data

Data files

- supplementary\_tables\_S1\_S3\_figure\_S1.pdf - Supplementary Data
