# Supplementary material for: Photosynthetic contribution of the ear to grain filling in wheat: a comparison of different methodologies for evaluation
Source: J Exp Bot. 2016 Mar 24;67(9):2787–98. doi: 10.1093/jxb/erw116 (PMC4861024; doi:10.1093/jxb/erw116)

***Journal of Experimental Botany* Supplementary Material**

Article title: *Photosynthetic contribution of the ear to grain filling in wheat: a comparison of different methodologies for evaluation*

Authors: Rut Sanchez-Bragado, Gemma Molero, Matthew P. Reynolds and Jose Luis Araus

The following supplementary material is available for this article:

TABLES

Table S1. Mean values of $\delta^{13}C$ in the WSF of the peduncle minus mature kernels ($\delta^{13}CWSF_P$-$\delta^{13}C_G$), the awns minus mature kernels ($\delta^{13}CWSF_A$-$\delta^{13}C_G$) and the flag leaf minus mature kernels ($\delta^{13}CWSF_F$-$\delta^{13}C_G$) after irrigation during the 2012 crop season. Differences in the $\delta^{13}C$ in the WSF after irrigation (AI) minus before irrigation (BI) in the peduncle ($\delta^{13}C\ WSF_{AI\text{-}P}$ - $\delta^{13}C\ WSF_{BI\text{-}P}$), awns ($\delta^{13}C\ WSF_{AI\text{-}A}$ - $\delta^{13}C\ WSF_{BI\text{-}A}$) and flag leaf ($\delta^{13}C\ WSF_{AI\text{-}F}$ - $\delta^{13}C\ WSF_{BI\text{-}F}$) during the 2012 crop cycle are also shown. Mean values are divided into two categories (see Materials and Methods); samples frozen immediately after sampling (frozen) and samples not frozen immediately after sampling (not frozen). Analysis of variance (ANOVA) for the sampling method effect (*P* values on the right column). Mean values with different superscripted letters are significantly different according to the Tukey's honestly significant difference test ($P<0.05$).

	**not-frozen**	**frozen**	**not-frozen/frozen**	***P***
$\delta^{13}C\ WSF_P$-$\delta^{13}C_G$	-2.10[a]	-1.63[b]	-0.47	0.02[*]
$\delta^{13}C\ WSF_A$-$\delta^{13}C_G$	-0.10[a]	0.44[b]	-0.54	0.049[*]
$\delta^{13}C\ WSF_F$-$\delta^{13}C_G$	-3.91[a]	-3.64[a]	-0.27	0.149[ns]
Average	*-2.03*	*-1.61*	*-0.43*	
$\delta^{13}C\ WSF_{AI\text{-}P}$ - $\delta^{13}C\ WSF_{BI\text{-}P}$	-1.56[a]	-0.91[b]	-0.65	0.02[*]
$\delta^{13}C\ WSF_{AI\text{-}A}$ - $\delta^{13}C\ WSF_{BI\text{-}A}$	-0.74[a]	-0.49[b]	-0.25	0.25[ns]
$\delta^{13}C\ WSF_{AI\text{-}F}$ - $\delta^{13}C\ WSF_{BI\text{-}F}$	-0.45[a]	-0.14[a]	-0.31	0.32[ns]
Average	*-0.91*	*-0.51*	*-0.40*	

Table S2. Values of $\delta^{13}C$ in the WSF of wheat leaves in the growth chamber experiment. Values are divided into two categories; samples frozen immediately after sampling and subsequently lyophilized (frozen) and samples not frozen and oven-dried (oven) after sampling. The correction factor was calculated from the difference in $\delta^{13}C$ in the WSF between leaves oven-dried six hours after sampling and leaves frozen with liquid nitrogen and subsequently lyophilized. Analysis of variance (ANOVA) for the sampling method.

	$\delta^{13}C$ WSF leaves (‰)		
	**Oven**	**Frozen**	**Frozen-Oven**
Rep. 1	-32.8	-32.1	0.7
Rep. 2	-31.8	-30.8	1.0
Rep. 3	-32.7	-31.9	0.9
Average	-32.5	-31.6	0.8
**Level of significance**			
Oven-Frozen	0.001**		

Table S3. Relative photosynthetic contributions of the ear and culm to grain filling assessed through the three methodologies considered in this study: (upper) DCMU treatment (2012 growing cycle) with DCMU application to the culm (DCMU culm) and the ear (DCMU ear); (middle) shading treatment (2013 growing cycle) with shaded culm and shaded ear and (lower) the $\delta^{13}C$ approach (2012 growing cycle BI and AI and 2013 growing cycle). In the first two methodological approaches (DCMU and shading) the percentage contributions (%) of the culms and ears were calculated relative to control (see Materials and Methods). The culm contribution (%) was calculated throughout the treatments where ear photosynthesis was inhibited either by DCMU application or by shading; the ear contribution (%) was calculated throughout the treatments where culm photosynthesis was inhibited, and the ear+culm contribution (%) represents the total sum of the ear and culm relative contributions. In the $\delta^{13}C$ approach, the relative (%) culm and ear contributions to grain filling were calculated as the proportion of the $\delta^{13}C_{peduncle}$ and $\delta^{13}C_{awns}$ of the WSF contributing to the $\delta^{13}C$ of mature kernels, respectively. The contribution of the ear considering the glumes (in addition of the awns) was also calculated using the $\delta^{13}C_{peduncle}$ and $\delta^{13}C_{awns+glumes}$ values of the WSF from samples taken in the 2012 crop cycle (2012 BI) and for the 2013 crop cycle. For all three approaches values presented are the averaged values ± standard deviation (SD) of the six bread wheat genotypes and three replicates per genotype. Grain weight per ear (GW_{ear}) and thousand kernel weight (TKW) were also calculated in the same six genotypes.

		Gw_{ear} (g)	(SD)	TKW (g)	(SD)	Ear contribution (%)	(SD)	Culm contribution (%)	(SD)	Ear+Culm contribution (%)	(SD)
**DCMU culm**											
Line pedigrees	CNO79//PF70354/MUS/3/PASTOR/4/BAV92*2/5/FH6-1-71	1.2	0.2	17.1	1.8	36.5					
	PBW343*2/KUKUNA*2//FRTL/PIFED2	1.7	0.6	25.6	7.8	72.2					
	SOKOLL//PBW343*2/KUKUNA/3/ATTILA/PASTOR3	1.1	0.3	22.3	4.9	43.0					
	TACUPETO F2001/BRAMBLING*2//KACHU4	1.1	0.4	16.8	3.1	37.4					
	UP2338*2/4/SNI/TRAP#1/3/KAUZ*2/TRAP//KAUZ/5/MILAN/KAUZ//CHIL/CHUM18/6/UP2338*2/4/SNI/TRAP#1/3/KAUZ*2/TRAP//KAUZ5	0.9	0.1	13.7	1.9	34.2					
	WBLL1*2/KURUKU*2/5/REH/HARE//2*BCN/3/CROC_1/AE.SQUARROSA(213)//PGO/4/HUITES6	1.7	0.7	23.2	12.6	49.0					
	Average	1.3	0.5	19.8	7.0	45.4	6.0				
**DCMU ear**											
Line pedigrees	CNO79//PF70354/MUS/3/PASTOR/4/BAV92*2/5/FH6-1-71	2.2	0.4	32.1	3.1			68.8			
	PBW343*2/KUKUNA*2//FRTL/PIFED2	1.7	0.3	27.8	3.9			69.4			
	SOKOLL//PBW343*2/KUKUNA/3/ATTILA/PASTOR3	1.9	0.1	34.8	0.9			72.2			
	TACUPETO F2001/BRAMBLING*2//KACHU4	2.0	0.1	31.5	1.5			65.9			
	UP2338*2/4/SNI/TRAP#1/3/KAUZ*2/TRAP//KAUZ/5/MILAN/KAUZ//CHIL/CHUM18/6/UP2338*2/4/SNI/TRAP#1/3/KAUZ*2/TRAP//KAUZ5	1.7	0.3	27.0	0.6			63.3			
	WBLL1*2/KURUKU*2/5/REH/HARE//2*BCN/3/CROC_1/AE.SQUARROSA(213)//PGO/4/HUITES6	2.5	0.3	36.6	3.5			73.1			
	Average	2.0	0.4	31.6	4.1			68.8	4.1		
**DCMU control**											
Line pedigrees	CNO79//PF70354/MUS/3/PASTOR/4/BAV92*2/5/FH6-1-71	3.2	0.2	45.5	0.2					105.2	
	PBW343*2/KUKUNA*2//FRTL/PIFED2	2.4	0.1	42.2	1.6					141.7	
	SOKOLL//PBW343*2/KUKUNA/3/ATTILA/PASTOR3	2.6	0.3	43.1	0.5					115.2	
	TACUPETO F2001/BRAMBLING*2//KACHU4	3.0	0.1	43.0	1.4					103.3	
	UP2338*2/4/SNI/TRAP#1/3/KAUZ*2/TRAP//KAUZ/5/MILAN/KAUZ//CHIL/CHUM18/6/UP2338*2/4/SNI/TRAP#1/3/KAUZ*2/TRAP//KAUZ5	2.6	0.1	40.3	1.2					97.5	
	WBLL1*2/KURUKU*2/5/REH/HARE//2*BCN/3/CROC_1/AE.SQUARROSA(213)//PGO/4/HUITES6	3.5	0.1	49.0	1.3					122.1	
	Average	2.9	0.4	43.9	3.0					114.2	16.1
**Shaded culm**											
Line pedigrees	BCN/RIALTO	1.1	0.2	20.5	1.4	57.1					
	BECARD/KACHU	1.7	0.2	29.5	2.8	72.0					
	SAUAL/4/CROC_1/AE.SQUARROSA(205)//KAUZ/3/ATTILA/5/SAUAL	1.5	0.0	28.6	2.1	52.8					
	CMH79A.955/4/AGA/3/4*SN64/CNO67//INIA66/5/NAC/6/RIALTO	1.9	0.2	27.6	1.8	66.1					
	KINGBIRD #1//INQALAB 91*2/TUKURU	1.2	0.0	25.9	3.2	59.0					
	WBLL1*2/KURUKU*2/5/REH/HARE//2*BCN/3/CROC_1/AE.SQUARROSA(213)//PGO/4/HUITES	1.6	0.3	31.4	5.2	52.8					
	Average	1.5		27.3		60.0	7.7				
**Shaded ear**											
Line pedigrees	BCN/RIALTO	1.3	0.1	24.6	1.4			67.2			
	BECARD/KACHU	1.4	0.3	31.0	6.3			60.0			
	SAUAL/4/CROC_1/AE.SQUARROSA(205)//KAUZ/3/ATTILA/5/SAUAL	1.7	0.1	33.4	4.5			59.8			
	CMH79A.955/4/AGA/3/4*SN64/CNO67//INIA66/5/NAC/6/RIALTO	1.9	0.2	27.9	2.3			66.6			
	KINGBIRD #1//INQALAB 91*2/TUKURU	1.2	0.1	25.4	1.4			57.1			
	WBLL1*2/KURUKU*2/5/REH/HARE//2*BCN/3/CROC_1/AE.SQUARROSA(213)//PGO/4/HUITES	1.5	0.2	39.1	7.4			48.4			
	Average	1.5	0.3	30.2	6.4			59.9	6.9		
**Shaded control**											
Line pedigrees	BCN/RIALTO	1.9	0.0	29.8	1.4					124.3	
	BECARD/KACHU	2.3	0.1	41.5	2.7					132.0	
	SAUAL/4/CROC_1/AE.SQUARROSA(205)//KAUZ/3/ATTILA/5/SAUAL	2.9	0.1	46.0	3.7					112.5	
	CMH79A.955/4/AGA/3/4*SN64/CNO67//INIA66/5/NAC/6/RIALTO	2.9	0.4	38.2	2.3					132.7	
	KINGBIRD #1//INQALAB 91*2/TUKURU	2.1	0.1	35.7	1.0					116.1	
	WBLL1*2/KURUKU*2/5/REH/HARE//2*BCN/3/CROC_1/AE.SQUARROSA(213)//PGO/4/HUITES	3.1	0.1	49.5	1.1					101.2	
	Average	2.5	0.5	40.1	7.0					119.8	12.2
**$\delta^{13}C$ approach**											
Interval $\delta^{13}C_{grain}$ (‰) — 2012 BI	[-25.2, -25.8]					75		25		100.0	
	[-25.8, -26.4]					50		50		100.0	
	[-26.4, -27.0]					25		75		100.0	
2012 AI	[-25.2, -25.8]					100		0		100.0	
	[-25.8, -26.4]					80		20		100.0	
	[-26.4, -27.0]					66		33		100.0	
2013	[-26.4, -27.0]					57		43		100.0	
	[-26.0, -26.4]					60		40		100.0	
	[-25.6, -26.0]					73		27		100.0	
	Average					65.1	21.0	34.7	21.0	100.0	
2012 BI +2013 (+glumes)	[-26.4, -27.0]					47		53		100.0	
	[-25.8, -26.4]					94		6		100.0	
	[-25.6, -25.8]					100		0		100.0	
	Average					80.3	29.0	19.7	29.0	100.0	

Figure S1. Daily mean precipitation (mm), evapotranspiration (mm) and air temperature (ºC) during the growing season from flowering to physiological maturity expressed as thermal time (ºC·day) during **(a)** the 2012 crop season and **(b)** the 2013 crop season. Vertical dotted lines symbolize sampling dates and vertical dashed lines represent dates of irrigation.

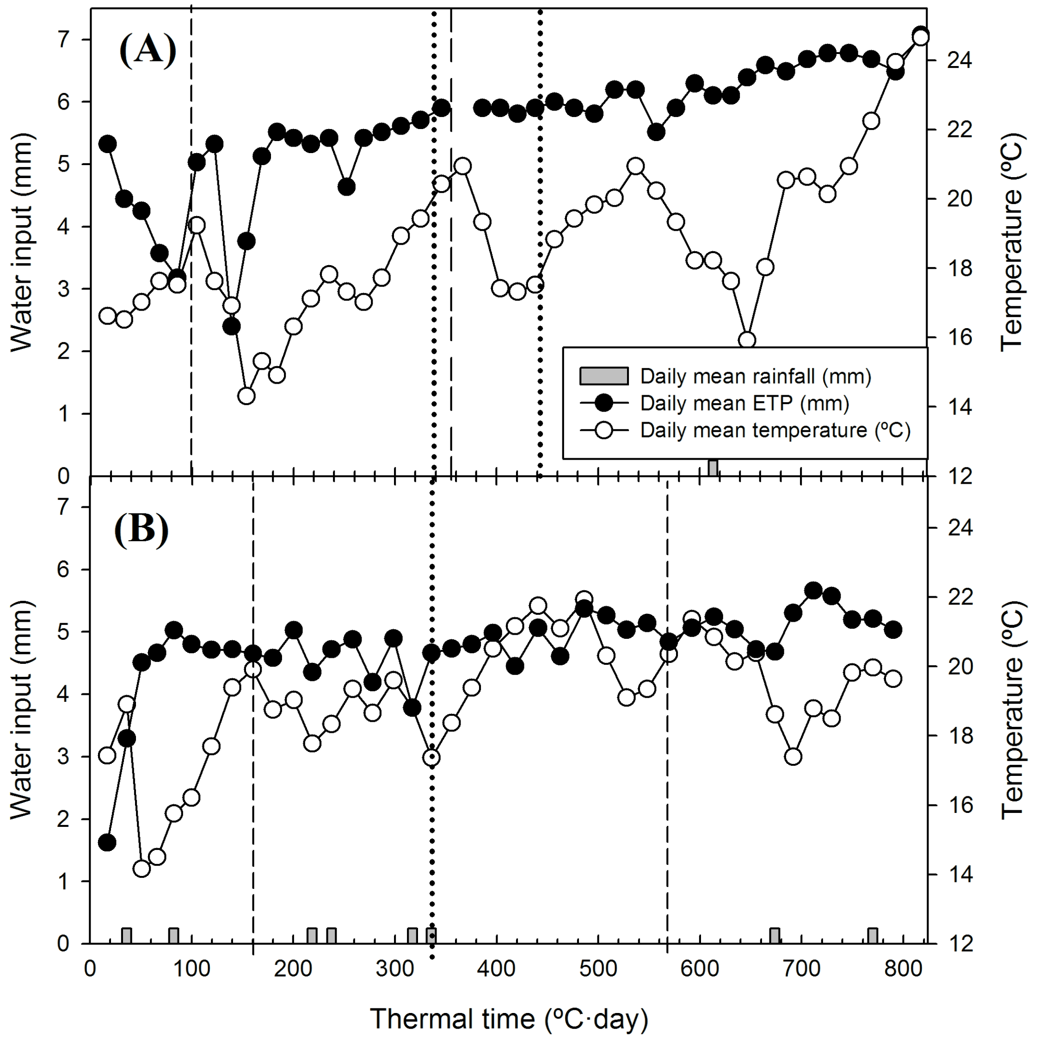

Supplement: Supplementary Data [file supp_erw116_supplementary_tables_S1_S3_figure_S1.pdf]
